# Corneal epithelium in keratoconus underexpresses active NRF2 and a subset of oxidative stress-related genes

**DOI:** 10.1371/journal.pone.0273807

**Published:** 2022-10-14

**Authors:** Tatiana Lupasco, Zhiguo He, Myriam Cassagne, Tomy Sagnial, Lise Brion, Pierre Fournié, Philippe Gain, Gilles Thuret, Michèle Allouche, François Malecaze, Michel Simon, Stéphane D. Galiacy

**Affiliations:** 1 Toulouse Institute for Infectious and Inflammatory Diseases (Infinity), Toulouse University, CNRS, Inserm, Paul Sabatier Toulouse III University, Toulouse, France; 2 Laboratory "Biology, Engineering, and Imaging of Corneal Graft", BiiGC, EA2521, Faculty of Medicine, Jean Monnet University, Saint-Etienne, France; 3 Department of Ophthalmology, Toulouse University Hospital, Toulouse, France; 4 Department of Ophthalmology, St Etienne University Hospital, Saint-Etienne, France; Cedars-Sinai Medical Center, UNITED STATES

## Abstract

Keratoconus (KC) is a multifactorial progressive ectatic disorder characterized by local thinning of the cornea, leading to decreased visual acuity due to irregular astigmatism and opacities. Despite the evolution of advanced imaging methods, the exact etiology of KC remains unknown. Our aim was to investigate the involvement of corneal epithelium in the pathophysiology of the disease. Corneal epithelial samples were collected from 23 controls and from 2 cohorts of patients with KC: 22 undergoing corneal crosslinking (early KC) and 6 patients before penetrating keratoplasty (advanced KC). The expression of genes involved in the epidermal terminal differentiation program and of the oxidative stress pathway was assessed by real time PCR analysis. Presence of some of the differentially expressed transcripts was confirmed at protein level using immunofluorescence on controls and advanced KC additional corneal samples. We found statistically significant under-expression in early KC samples of some genes known to be involved in the mechanical resistance of the epidermis (*KRT*16, *KRT14*, *SPRR1A*, *SPRR2A*, *SPRR3*, *TGM1* and *TGM5*) and in oxidative stress pathways (*NRF2*, *HMOX1* and *HMOX2*), as compared to controls. In advanced KC samples, expression of *SPRR2A* and *HMOX1* was reduced. Decreased expression of keratin (KRT)16 and KRT14 proteins was observed. Moreover, differential localization was noted for involucrin, another protein involved in the epidermis mechanical properties. Finally, we observed an immunofluorescence staining for the active form of NRF2 in control epithelia that was reduced in KC epithelia. These results suggest a defect in the mechanical resistance and the oxidative stress defense possibly mediated via the NRF2 pathway in the corneal keratoconic epithelium.

## Introduction

The corneal epithelium displays some similarities with the epidermis. Both are multilayer polarized epithelia derived from the ectoderm and form, at the surface of eye or skin, a multifunctional barrier that protects from harmful agents, such as microbes, chemicals and UV radiations. In the epidermis, most of these functions are provided by the upper cornified layer. This layer is also called *stratum corneum* and characterizes the ultimate step of the keratinocyte differentiation program. In contrast, the corneal epithelium lacks a cornified layer. However, in rare corneal diseases some degree of cornification has been reported [1, 2]. A shift of the corneal epithelium toward an epidermis like phenotype has been found in mouse models of dry eye [3, 4].

Keratoconus (KC) is a progressive ectatic disease characterized by progressive thinning and deformation of the cornea, causing corneal protrusion, irregular astigmatism and decreased visual acuity. Eventually, corneal deformation and associated mechanical stress may result in irreversible corneal opacities. KC is the most common corneal ectasia with an incidence of 1 in 2000 in the general population [5]. When visual acuity can no longer be corrected by contact lenses or intracorneal rings, the only treatment consists in cornea transplantation. The etiology of KC is unknown and suspected to be multifactorial, with the involvement of genetic and environmental factors [6]. To date, several genes have been associated with this disease [7], including, but not limited to, those of lysyl oxidase, interleukin-1 [8], visual system homeobox 1 [9], microRNA 184 [10], transforming growth factor beta [11], and zinc finger protein 469 [12]. Allergy, either ocular or general [13] (spring keratoconjunctivitis and atopic diseases), mechanical microtrauma induced by contact lens and eye rubbing, and corneal thinning following Laser-Assisted In-Situ Keratomileusis surgery are thought to be involved in the disease progression and development.

KC is a complex disorder and appears to be the manifestation of various pathological processes: tissue disruption impacting epithelial basal lamina and Bowman’s layer [5], keratocyte apoptosis [14], enzymatic imbalances [15], inflammation [16], oxidative stress [17], autophagy [18] and hormonal changes [19]. Recent studies support a chronic inflammatory process [20], as well as a possible link with atopic dermatitis [21] in the pathogenesis of the disease. While the stroma might be the essential layer of the disease development, corneal epithelium could also play a secondary and preliminary role. Indeed, in keratoconic eyes, the corneal epithelium presents a localized thinning at the level of the cone apex surrounded by a thickening ring with a kind of donut pattern that is highly characteristic of this condition [22]. In KC, both epithelial hyperplasia and hypoplasia have been described, associated or not with a rupture of the basal lamina and Bowman’s layer. It has been suggested that the KC epithelium undergoes a remodeling process to mechanically compensate the abnormalities of the stromal surface [23]. However, epithelium cornification has never been reported. We previously identified a number of differentially expressed genes in whole cornea of KC, suggesting an imbalance between apoptosis and cellular proliferation and differentiation [24].

Based on these arguments, we hypothesized that the corneal epithelium could be involved in early KC development. The aim of this study was to analyze the expression of genes related to the epidermal terminal differentiation program in corneal epithelium of KC patients compared to controls.

## Materials and methods

### Biological material

Control epithelium samples (CTRL group) were collected from patients undergoing Photo Refractive Keratectomy procedure for mild myopia (less than 3 diopters): prior to the Excimer laser ablation, cornea was prepared by manual removal of the central (8 mm diameter) epithelium. The epithelium was scraped off with Beaver-type blade, collected in a sterile RNAse-free eppendorf, and immediately immersed in liquid nitrogen for transport. Before RNA extraction, the samples were stored at -80°C. Corneal epitheliums from patients with progressive KC (early KC group) were collected during corneal crosslinking procedure used to halt the disease progression. Manual de-epithelialization is necessary for the impregnation of the corneal stroma with riboflavin before irradiation with UVA. The samples were thus collected, transported and stored with the same conditions as above. The third group of corneal epithelium samples (advanced KC, stage IV i.e. with corneal opacities) was similarly obtained from patients undergoing penetrating keratoplasty surgery. While samples were from different surgical conditions, they were collected using the same technical procedure performed by the same surgeon. Average age was 28.5 ± 5.7 years in the CTRL group, 22 ± 4.2 years in the early KC group, and 45 ± 7 years in the advanced KC group. Gender distribution was 63.6% men in CTRL group and 68.2% in KC group. 40% and 50% respectively of CTRL and KC group patients had history of allergies and/or atopy. 13.6% of KC group patients were using hard contact lens.

For immunofluorescence (IF) analysis, 9 fresh corneas were used as healthy epithelial controls. They came from 9 body donation for science. Each donor volunteered their body and gave written consent to the Laboratory of Anatomy of Jean Monnet University. The fresh corneas were included immediately in Tissue-Tek O.C.T. tissue embedding medium after dissection from the eyeballs, and then stored at -80°C. The mean donor age was 76.6 ±4.5 years. The time between death and tissue embedding was 17.3±2.6 hours. 3 KC corneas were collected during corneal graft surgery with an average age at collection of 44±6 years. Samples were placed in O.C.T. and immediately frozen in liquid nitrogen then kept at -80°C until further use.

Human cornea epithelial samples used in this study were obtained from the Department of Ophthalmology and from the French National Reference Center for Keratoconus, Toulouse University Hospital between 2014 and 2019. All procedures were in accordance with the Declaration of Helsinki of 1975 and its 1983 revision in protecting donor confidentiality. Informed consent was obtained from all participants prior to sample collection with authorization from the Patient Protection Committee (DC2012-1701).

### Reverse transcriptase real time polymerase chain reaction

Total RNA was extracted with Qiagen MicroRNA extraction kit according to the manufacturer’s instructions (Qiagen S.A.S., Cortaboeuf, France). Reverse transcriptase was performed using the Invitrogen Superscript III VILO kit according to the manufacturer’s recommendations (ThermoFisher Scientific, Villebon-sur-Yvette, France). Real time polymerase chain reaction (RT-PCR) was performed on triplicate samples (50 pg cDNA) in a Roche LightCycler 480 using Roche supermix for PCR (Roche, Paris, France). Expression of 22 genes involved in epidermal terminal differentiation, 6 genes involved in Nuclear factor erythroid 2-related factor 2 (*NRF2*) pathway, and of keratin 3 encoding gene (*KRT3)* as a tissue specific gene of the corneal epithelium was investigated. We selected some markers of early and late keratinocyte differentiation, based on our previous work [25]. Primer sequences are described in S1 Table. The PCR conditions were: 95° C for 10 min, followed by 40 cycles of denaturation at 95° C for 15 s and annealing at 60° C for 20 s. We used three genes for normalization by geometric averaging (encoding ubiquitin, TATA-box binding protein and beta 2 macroglobulin) determined by a method previously described [25]. Fold-change in gene expression was calculated using the 2-^ΔΔCt^ ratio according to the previously described method [26]. All PCR products were checked by sequencing and the primer efficiencies were examined using cDNA dilutions (from 1/10 to 1/320) and Roche software analysis program.

### Immunofluorescence (IF)

As previously described [27], corneal sections (10 μm thick) were prepared using Cryostat Microm HM550 (ThermoFisher Scientific). Frozen slides were warmed and dried at RT for 5 min then rehydrated for 5 min. Samples were then incubated at 37°c for 30 min in saturating solution (PBS supplemented with 2% heat-inactivated goat serum (Eurobio-Ingen, Les Ullis, France) and 2% bovine serum albumin (ThermoFisher Scientific)). Slides were incubated at 37°C for 1 hour with primary antibodies (anti-involucrin (IVL) clone SY5, Sigmal-Aldrich 1:400; anti-KRT3 clone AE5, Merk-Millipore 1:400; anti-KRT 16 clone LL025, Thermofisher 1:400; anti-KRT14 clone LL002, Merk-Millipore 1:400, anti-NRF2(phosphoS40) ab76026, Abcam 1:400, anti-HMOX1 HPA000635, Sigma-Aldrich 1:400) diluted in saturating solution. Nonspecific rabbit and mouse IgG (Zymed, Carlsbad, CA, USA) were used as primary antibodies for negative controls. These two controls were performed for each cornea. Secondary antibodies were Alexa Fluor 488 goat anti-mouse or anti-rabbit IgG for single staining and Alexa Fluor 488 goat anti-mouse combined with Alexa Fluor 555 goat anti-rabbit IgG (Invitrogen, Eugene, OR, USA) for double staining. Secondary antibodies diluted by 1:1000 in saturating solution were incubated for 1 hour at 37°C. Lastly, nuclei were counterstained with TO-PRO-3 Iodide (1/1000) (ThermoFisher Scientific) in PBS for 5 min at RT. Three rinses in PBS were performed between each step except between blocking of nonspecific protein binding sites and incubation with primary antibodies. Finally, the slides were mounted using Vectashield medium (Vector Laboratories, Burlingame, CA, USA). Images were captured with a confocal microscope (IX83 Fluoview FV-1000, Olympus), equipped with the Olympus Fluoview software. Setup were determined from the control conditions and applied to the other samples.

### Statistical analyses

For all experiments group to group comparisons were performed and plotted with GraphPad Prism 6 using a non-parametric Kruskal-Wallis test. *P* < 0.05 was considered as statistically significant.

## Results

### Differentially expressed transcripts in KC versus control corneal epithelium

The corneal epithelium of 23 CTRL and 28 KC patients (22 with a progressive form of the disease (early KC) and 6 with corneal opacities (advanced KC)) was collected. Gene expression was analyzed using real time PCR.

We first evaluated the expression of 22 genes involved in epidermal terminal differentiation, and of *KRT3* as a typical gene of the corneal epithelium.

In the CTRL group, high expression of *KRT3* was observed (Table 1). Concerning the epidermal differentiation genes, three classes of transcripts based on their relative abundance to normalization genes could be observed in the CTRL group (Table 1). Highly expressed genes were those encoding desmoglein 1 (*DSG1)*, claudin 1 (*CLDN1)*, desmoplakin *(DSP)*, keratin *14 (KRT14)* and periplakin *(PPL)* (proteins of desmosomes, keratin filaments and tight junctions). Medium expressed genes were those encoding desmoglein 2 (*DSG2)*, desmoglein 3 (*DSG3)* and envoplakin (*EVPL)* (proteins of desmosomes); small proline-rich protein 2A *(SPRR2A)*, involucrin *(IVL)*, small proline-rich protein 1A (*SPRR1A)*, small proline-rich protein 3 (*SPRR3)*, four proteins of the epidermis cornified envelope, a peculiar pericellular structure of cornified cells that replaces plasma membrane; transglutaminase 1 (TGM1) and transglutaminase 5 (TGM5), two enzymes involved in cornified envelope formation; and keratin 16 (*KRT16)*. Finally, poorly or not expressed genes were those of filaggrin (*FLG)*, hornerin (*HRNR)*, loricrin (*LOR)*, desmocollin 1 (*DSC1)*, corneodesmosin *(CDSN)*, filaggrin 2 (*FLG2)*, transglutaminase *3 (TGM3)* and keratin 10 (*KRT10)*.

**Table 1:** Transcript relative expression of epidermal terminal differentiation genes in control corneal epithelium.

Genes Name	dCT*	SEM†
*KRT3*	-4.97	0.14
*DSG1*	-2.67	0.14
*CLDN1*	-1.04	0.11
*DSP*	-0.97	0.14
*PPL*	-1.09	0.17
*DSG2*	1.06	0.25
*KRT14*	2.44	0.34
*SPRR2A*	3.55	0.37
*DSG3*	3.78	0.84
*EVPL*	3.79	0.19
*TGM1*	3.91	0.17
*IVL*	4.69	0.17
*SPRR1A*	4.44	0.69
*KRT16*	5.12	0.19
*TGM5*	5.32	0.52
*SPRR3*	6.57	0.90
*FLG*	8.23	0.26
*HRNR*	9.96	0.28
*LOR*	10.08	0.34
*DSC1*	Nd	
*CDSN*	Nd	
*FLG2*	Nd	
*TGM3*	Nd	
*KRT10*	Nd	

* Mean expression normalized to that of housekeeping genes (geometric mean of *UBB*, *TBB* and *B2M*). dCT: deltaCycleThreshold. † Standard error to the mean. Nd: not detected.

**Fig 1:**
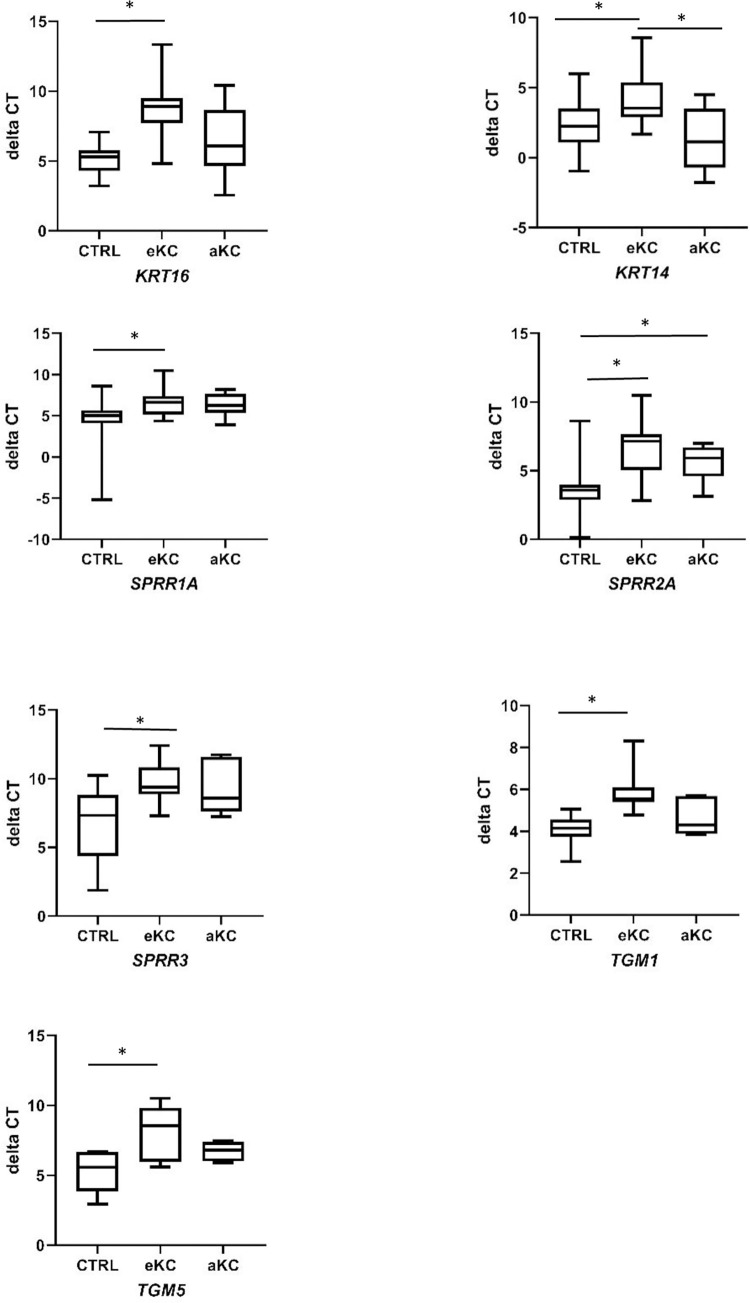
Differentially expressed transcripts in early and advanced keratoconus. Box and whiskers plots of gene expression in the corneal epithelium of control (CTRL; n = 23), early keratoconus (eKC; n = 22) and advanced keratoconus (aKC; n = 6). Transcript level expressed as mean of delta cycle threshold (dCT) normalized to housekeeping genes. Non-parametric Kruskal-Wallis test. *P* < 0.05 was considered as statistically significant.*, P value < 0.05.

We then compared the expression of this set of genes between CTRL and early KC groups (Fig 1, Table 2), considering as differentially expressed, genes with a fold change either ≥ 2 or ≤ 0.5 with a *P* value ≤ 0.05. We observed in the early KC a significant decrease in the expression of *KRT16*, *KRT14*, *SPRR1A*, *SPRR2A*, *SPRR3*, *TGM1* and *TGM5* (fold change of 0.09, 0.31, 0.21, 0.11, 0.10, 0.27 and 0.14, respectively, P value of <0.0001, 0.044, 0.0022, <0.0001, 0.0006, <0.0001 and 0.009, respectively). We performed partial subset analysis comparing atopic/allergic to non-atopic/allergic individuals (number of samples was low for some subgroups), without finding major differences of expression in these subgroups compared to the general analysis (S2 Table).

**Table 2:** Genes with significant differential transcript expression in early and advanced keratoconus.

Gene Name	CTRL (dCT)* ±$	eKC (dCT)* ±$	Fold Change	p value	aKC (dCT)* ±$	Fold Change	p value
*KRT16*	5.12±0.19	8.61±0.38	0.09	<0.0001	6.42±1.09	0.41	0.5125
*KRT14*	2.44±0.34	4.11±0.42	0.31	0.044	1.30±0.97	2.20	1
*SPRR1A*	4.44±0.69	6.72±0.3734	0.21	0.0022	6.32±0.61	0.25	0.056
*SPRR2A*	3.55±0.37	6.74±0.46	0.11	<0.0001	5.61±0.59	0.22	0.011
*SPRR3*	6.57±0.9079	9.89±0.31	0.10	0.0006	9.24±0.79	0.13	0.11
*TGM1*	3.91±0.17	5.81±0.29	0.27	<0.0001	4.62±035	0.69	0.086
*TGM5*	5.32±0.52	8.19±0.54	0.14	0.009	6.76±0.27	0.37	0.081

* Mean expression normalized to that of housekeeping genes (geometric mean of *UBB*, *TBB* and *B2M*). dCT: deltaCycleThreshold.

^$^ Standard error to the mean. CTRL, control; eKC, early keratoconus; aKC, advanced keratoconus. Differentially expressed genes (0.5 ≥ fold change ≥ 2 and P value ≤ 0.05) are indicated in grey. Non-parametric Kruskal-Wallis test. *P* < 0.05 was considered as statistically significant.

The same analysis was then performed in advanced KC (Fig 1 and Table 2). A decreased expression of *SPRR2*A was observed compared to CTRL (fold change 0.24, P value of 0.011). A tendency of decreased expression of *SPRR1A*, *SPRR3*, *TGM1* and *TGM5* was noted, but statistical significance was not reached.

### Differentially expressed proteins

**Fig 2:**
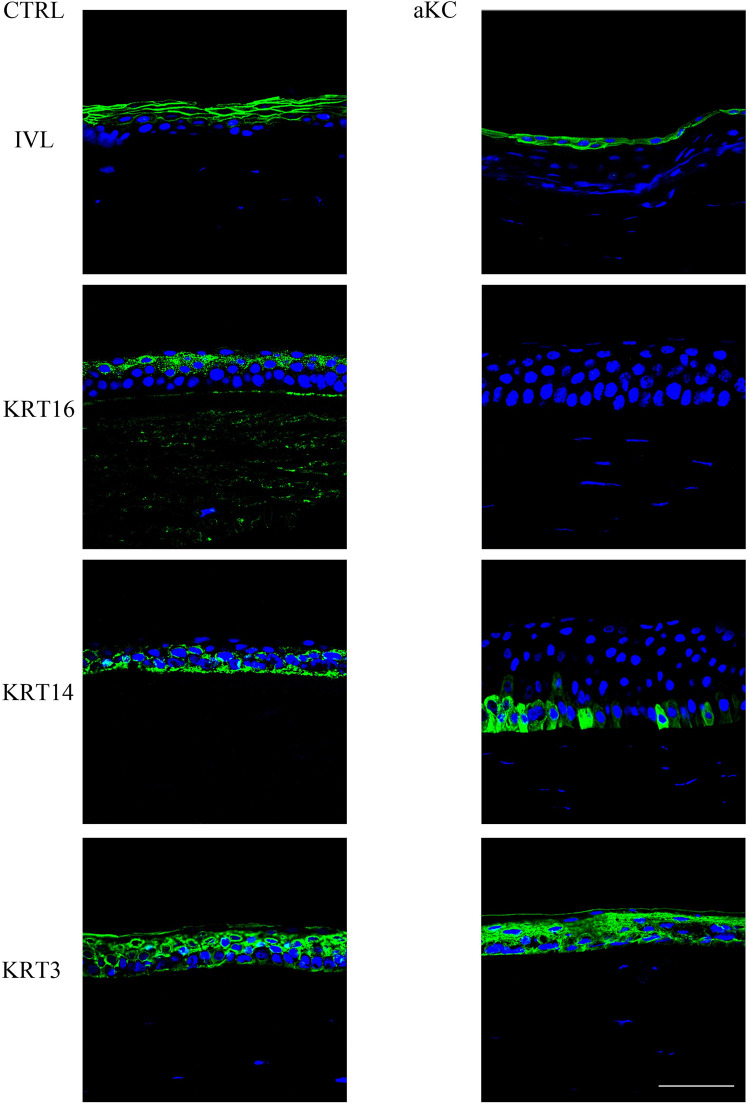
Differentially expressed proteins in advanced KC. Sections of normal (CTRL; n = 3) and advanced keratoconus (KC; n = 3) corneas were analyzed by indirect immunofluorescence (green) with the indicated antibodies. Nuclei were stained with TO-PRO Iodide (blue). Scale bar = 100 μm.

We then analyzed, by confocal microscopy, the immunofluorescence staining of several antibodies targeting proteins for which we observed a variation in mRNA expression (Fig 2). We first observed, as expected [28], differences in the KC epithelial thickness on the same section with areas of hyperplasia and areas of hypoplasia (see S1 Fig). In the CTRL group, IVL was highly detected in the upper cell layers of the epithelium (apical and underlying intermediate). In the advanced KC group, IVL was detected only in the most apical cell layer.

KRT16 was detected in the upper epithelial cell layers of the CTRL group whereas it was not or barely detected in the advanced KC group.

KRT14 was found all over the cornea and especially in the basal layers of control samples, while we observed a signal disappearance in KC samples from the periphery toward central cornea (S2 Fig).

Finally, KRT3 was strongly expressed in the corneal epithelium of both groups without any visible differences.

### Decreased expression of genes related to oxidative stress resistance

**Fig 3:**
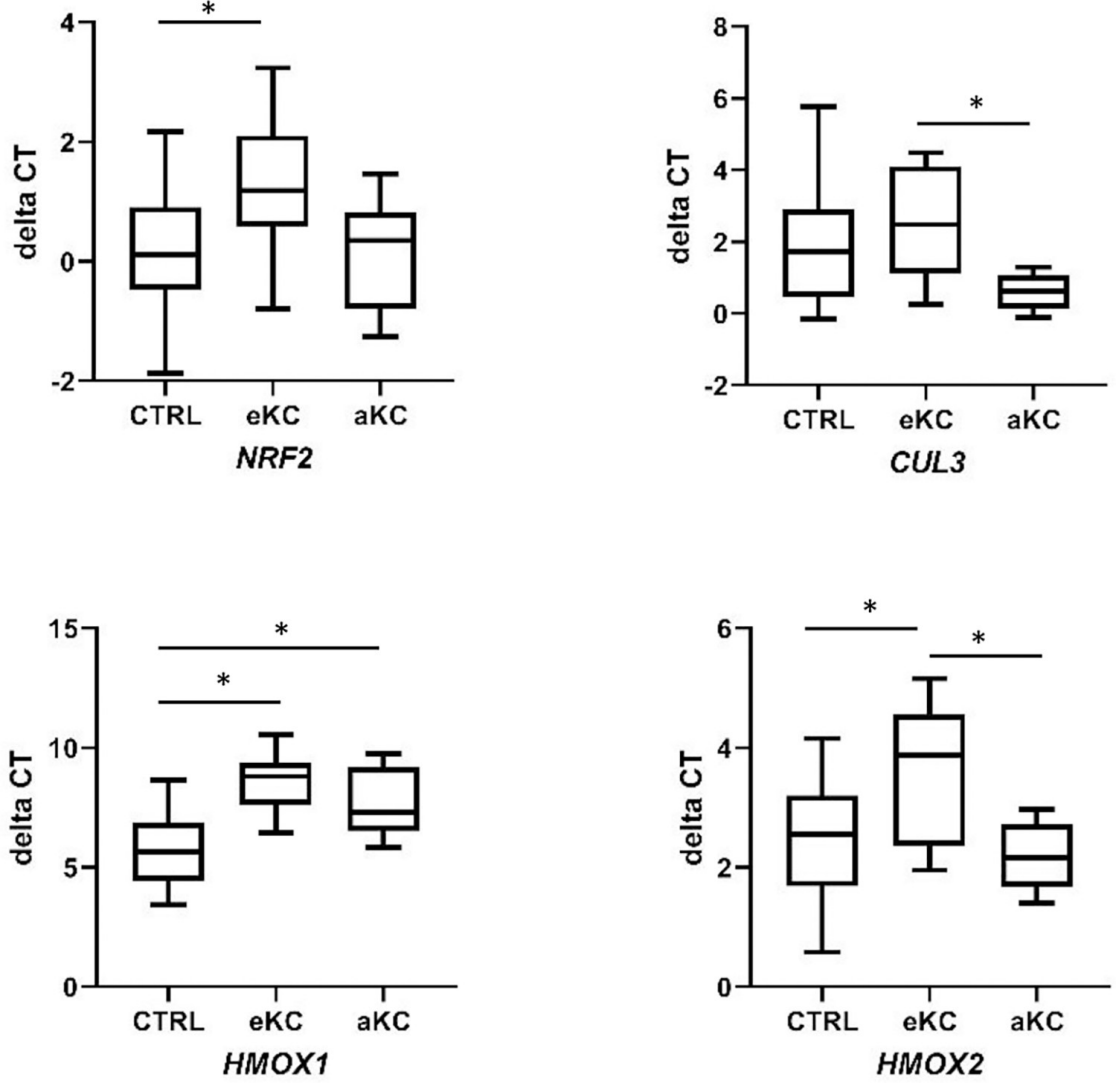
Comparative expression of NRF2 pathway genes in early and advanced keratoconus. Box and whiskers plots of gene expression in the corneal epithelium of control (CTRL; n = 20), early keratoconus (eKC; n = 11) and advanced keratoconus (aKC; n = 6). Transcript level expressed as mean of delta cycle threshold (dCT) normalized to housekeeping genes. Non-parametric Kruskal-Wallis test. *P* < 0.05 was considered as statistically significant.*, P value < 0.05.

In early KC, we observed a decreased expression of *NRF2* mRNA (fold change of 0.46, P value of 0.0486). NRF2 target *HMOX*1 mRNA, encoding heme oxygenase 1, was also strongly underexpressed in early and advanced KC samples (fold change of 0.14 and 0.27, respectively; P value of <0.0001 and 0.016 respectively), while *HMOX2* mRNA was only underexpressed in early KC vs CTRL (fold change of 0.41, P value of 0.012). Other genes encoding either regulators (Cullin 3 (*CUL3)* and Kelch-like ECH-associated protein 1 (*KEAP1)*) or targets (*NQO1* encoding NAD(P)H deshydrogenase (quinone) 1) of NRF2 had a similar expression in both groups (Fig 3 and Table 3).

**Table 3:** Transcript relative expression of NRF2 pathway genes in control and keratoconus corneal epithelium.

Genes Name	CTRL (dCT*±$)	dCT eKC*	Fold change	P value	dCT aKC*	Fold change	P value
*NRF2*	0.17±0.24	1.3±0.38	0.46	0.028	0.15±0.39	1.03	1
*CUL3*	1.77±0.37	2.63±0.49	0.55	0.147	0.61±0.21	1.75	0.4533
*KEAP1*	4.17±0.3526	4.54±0.34	0.77	0.57	3.59±0.26	1.54	0.605
*HMOX1*	5.77±0.36	8.61±0.35	0.14	<0.0001	7.66±0.59	0.25	0.0317
*HMOX2*	2.39±0.24	3.66±0.34	0.41	0.012	2.18±0.25	1.29	0.709
*NQO1*	-3.25±0.18	-3.33±0.13	1.06	0.58	-3.40±X	0.97	0.55

* Mean expression normalized to that of housekeeping genes. dCT: deltaCycleThreshold.

^$^ Standard error to the mean. CTRL, control; eKC, early keratoconus; aKC, advanced keratoconus. Differentially expressed genes (0.5 ≥ fold change ≥ 2 and P value ≤ 0.05) are indicated in grey. Non-parametric Kruskal-Wallis test. *P* < 0.05 was considered as statistically significant.

**Fig 4:**
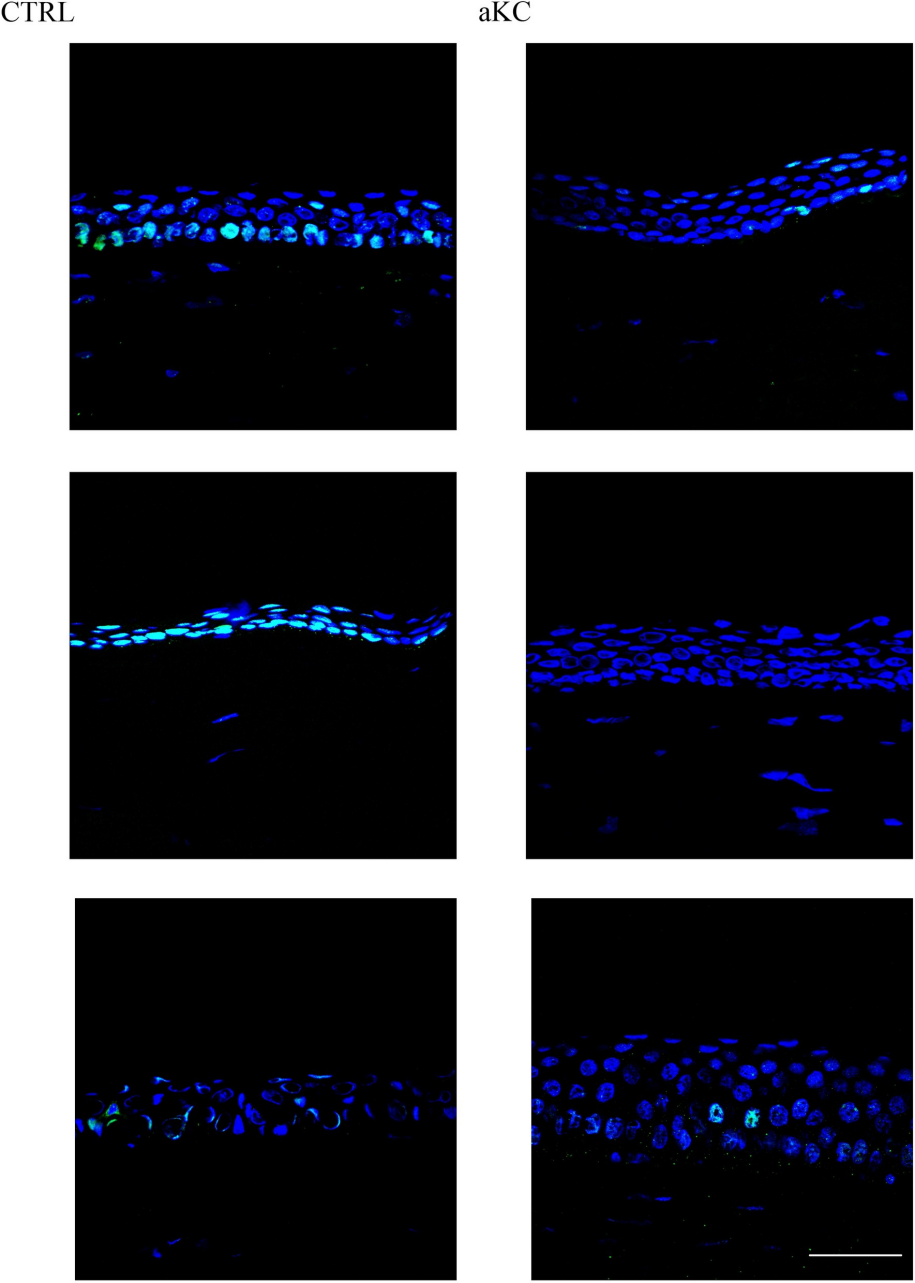
NRF2 active form staining in human corneal material. Sections of normal (CTRL; n = 5) and advanced keratoconus (KC; n = 3) corneas were analyzed by indirect immunofluorescence with an antibody targeting the active phosphorylated form of NRF2 (green). Nuclei were stained with TO-PRO Iodide (blue). Scale bar = 100 μm. Figure show three representative controls and keratoconus.

Immunofluorescence staining produced by an antibody targeting the active form of NRF2, i.e., the S40 phosphorylated NRF2, revealed a strong expression in control material with a nuclear localization as expected (Fig 4). In KC material, phosphorylated NRF2 staining was reduced with only a few cells stained in their nucleus (Fig 4).

## Discussion

In our previous transcriptome analysis of KC versus normal whole corneas, we reported that in KC there was an imbalance in apoptosis, proliferation and differentiation pathways. In this work, we hypothesized that corneal epithelium might have a more important role in KC development that what is actually described. Thus, we decided to investigate corneal epithelium transcripts of KC patients when the KC is evolutive and before major stromal changes (when stromal depth is still superior to 400μm), patients we named “early KC”. As little is known about the terminal differentiation program of the corneal epithelium, we based our investigation on epidermal terminal differentiation genes as epidermis is better described and shares some common feature with cornea. Transcript expression analysis revealed that some but not all keratinocyte terminal differentiation genes are also expressed in the normal cornea epithelium. Not surprisingly, most of the cornification related genes were not or barely detected (*FLG*, *FLG2*, *LOR*, *HRNR*, *CDSN*, *DSC1* and *TGM3*). Interestingly, we detected a set of transcripts (*IVL* and *SPRRs*) encoding proteins that, in the epidermis, are part of cornified envelope. This is a resistant highly cross-linked structure that replaces the plasma membrane of corneocytes, the end products of keratinocyte differentiation. IVL protein expression was confirmed by immunohistochemistry (S3 Fig). Our results are in agreement with those of Tong et al. [29]. We also detected mRNAs encoding the enzymes necessary for cornified envelope formation, namely transglutaminases 1 and 5. However, cornified envelopes appear to be absent in the corneal epithelium. Indeed, we were not able to observe them after applying to corneal epithelium the procedure currently used to purify epidermal cornified envelopes [30], (S4 Fig). Thus, the role of IVL and SPRRs in the cornea is not clear. They may be associated with cell mechanical resistance properties as previously suggested [29]. As we found these transcripts underexpressed in KC corneas, this could suggest that biomechanical properties of epithelial cells are impaired in KC. In agreement to this statement, we observed decreased expression of other structural proteins, i.e., intermediate filament components KRT16 and KRT14, accordingly to mRNA variation. Little is known about KRT16 function in the cornea. KRT16 has been found to be overexpressed in epidermis diseases in hyperproliferative areas [31] and during corneal wound healing [32]. Whether KRT16 expression is associated to the proliferative state of the cell or to the loss of phenotype remains unclear [32]. Two previous proteomic analyses have reported an overexpression of KRT16 in KC corneas [33, 34]. The discrepancy between this and our results could be explained by differences in disease severity [35]. Moreover KRT14 was also underexpressed. KRT14 is often associated with mitotic active cells [36] both in the epidermis and the cornea.

However, as a number of mechanical partners of IVL and SPRRs are missing in the corneal epithelium, this leads us to consider alternative function for these genes in the cornea. Indeed, some SPRRs are able to quench reactive oxygen species [37–39] thus we propose that they may display oxidative stress protecting properties in the cornea. Moreover, SPRRs promoter contain an AP1 response element, which could explain their reduced expression in KC corneas as we and others have previously demonstrated [24, 40], that this transcription factor is underexpressed in KC corneas. Some SPRRs genes are also under the control of NRF2 transcription factor [37]. NRF2 is known to target several anti-oxidative stress enzymes, namely HMOX1, HMOX2 and NQO1, that we found to be also expressed in the corneal epithelium. We then compared the expression levels of these transcripts between CTRL and early KC corneal epithelium. We here described for the first time that in KC epithelium, transcripts of *NRF2* and its target genes *HMOX*1 and *HMOX2* are downregulated. These data suggest an increased sensitivity of KC corneal epithelium to oxidative stress. HMOX enzymes are important as they mediate the first step of heme catabolism [41]. Iron deposition and reduced oxidative stress response are observed in *Hmox*1 knock-out mice [42]. Interestingly iron accumulation is frequently observed in KC corneas [43]. Moreover, HMOX1 promoter also contains an AP1 response element, which is the other pathway used to activate this enzyme. Unfortunately, we have not been able so far to evidence HMOX1 protein in our material. More work is needed to clearly validate HMOX1 as an interesting candidate. A recent RNA sequencing study by Shinde et al. also demonstrated an involvement of NRF2 pathway [44]. While the authors did not observed a variation of NRF2 mRNA itself, they identified changes in the NRF2 pathway by gene ontology analysis and confirmed NRF2 underexpression at the protein level in KC corneas. Similar to our work, they found some targets of NRF2 to be underexpressed in KC samples [44]. Moreover, several recent studies support these findings. Stachon et al found that primary culture of stromal fibroblasts from KC patient were not able to increase anti-oxidant pathways following hypoxic stress [45]. Lopez-Lopez et al. in a tear proteomics study observed a decreased expression of proteins involved in iron homeostasis and inflammatory response [46]. In this context, it is interesting to note that in mouse, sulforaphane-induced increase in KRT16 expression is dependent on NRF2 [47]. Whether the strong inhibition of KRT16 that we noted is related to the down regulation of NRF2 remains to be tested. More work is needed to characterize downstream regulation of NRF2. A recent review pointed out all the targets already investigated in KC with different techniques [48]. However for all targets results have conflicted (up or down regulation depending of the studies). In our study, we bring a new brick as we showed that NRF2 active form was expressed in CTRL epithelia while it was not or barely detectable in KC epithelia.

Analysis in advanced KC mRNA did not provide a lot of useful data, while IF was more convincing. Only *HMOX1* mRNA was found underexpressed. We hypothesized that it might be due to a lack of power. As these materials are coming from less and less used surgery procedure, it will take longer time to pursue these investigations on advanced KC mRNA.

All these data strongly suggest that in KC epithelia, several pathways that are working together to protect the cornea from oxidative stress and mechanical injury are severely impaired in early stages. Functional investigations are now required for understanding whether this impairment is directly due to KC epithelium defects or is indirectly driven by others sources (tears, nerves, stroma, etc.).

## Supporting information

S1 Fig Variation in epithelial thickness on the same section of the corneal epithelium in keratoconus. Representative immunodetection of KRT3 (green) on frozen sections of human corneal epithelium of keratoconus patients (n = 3). Nuclei were stained with TO-PRO Iodide (blue). A-C, Enlargements of the indicated areas. A: peripheral hyperplasic zone, B: intermediate zone, normal thickness, C: corneal apex hypoplasic zone.
(TIF)

S2 Fig Variation in KRT14 expression in the corneal epithelium in keratoconus. Representative immunodetection of KRT14 (green) on frozen sections of human corneal epithelium of keratoconus versus control patients. Nuclei were stained with DAPI (blue). A: control patient; B: keratoconus patient.
(TIF)

S3 Fig Involucrin (IVL) protein expression. Proteins of control (CTRL) and early keratoconus (eKC) samples were immunoblotted with antibodies specific for IVL and actin, respectively (one representative blot with 4 samples of each condition). Densitometry quantification of western-blots using Image J; n = 12, P = 0.27.
(TIF)

S4 Fig Cornified envelopes extraction from human abdominal epidermis and human corneal epithelium. Optical microscope observation. X20 magnification.
(TIF)

S1 Methods Immunoblotting analysis. (DOCX)

S1 Table Sequences of primers used for qPCR. (DOCX)

S2 Table Differential expression analysis of different subgroups of patient. (DOCX)

S1 Raw images (PDF)

## References

[1] Pflugfelder SC, Stern ME. The cornea in keratoconjunctivitis sicca. Exp Eye Res. 2020 Dec;201:108295. doi: 10.1016/j.exer.2020.108295 . Pubmed Central PMCID: 7736263. 33038387 PMC7736263

[2] Soler VJ, Tran-Viet KN, Galiacy SD, Limviphuvadh V, Klemm TP, St Germain E, et al. Whole exome sequencing identifies a mutation for a novel form of corneal intraepithelial dyskeratosis. J Med Genet. 2013 Apr;50(4):246–54. doi: 10.1136/jmedgenet-2012-101325 . Pubmed Central PMCID: PMC4115150. 23349227 PMC4115150

[3] Corrales RM, de Paiva CS, Li DQ, Farley WJ, Henriksson JT, Bergmanson JP, et al. Entrapment of conjunctival goblet cells by desiccation-induced cornification. Invest Ophthalmol Vis Sci. 2011 Jun 1;52(6):3492–9. doi: 10.1167/iovs.10-5782 . Pubmed Central PMCID: 3109039. 21421863 PMC3109039

[4] De Paiva CS, Villarreal AL, Corrales RM, Rahman HT, Chang VY, Farley WJ, et al. Dry eye-induced conjunctival epithelial squamous metaplasia is modulated by interferon-gamma. Invest Ophthalmol Vis Sci. 2007 Jun;48(6):2553–60. doi: 10.1167/iovs.07-0069 . 17525184

[5] Kenney MC, Nesburn AB, Burgeson RE, Butkowski RJ, Ljubimov AV. Abnormalities of the extracellular matrix in keratoconus corneas. Cornea. 1997 May;16(3):345–51. . 9143810

[6] Gordon-Shaag A, Millodot M, Shneor E, Liu Y. The genetic and environmental factors for keratoconus. Biomed Res Int. 2015;2015:795738. doi: 10.1155/2015/795738 . Pubmed Central PMCID: PMC4449900. 26075261 PMC4449900

[7] Hao XD, Chen P, Chen ZL, Li SX, Wang Y. Evaluating the Association between Keratoconus and Reported Genetic Loci in a Han Chinese Population. Ophthalmic Genet. 2015 Jun;36(2):132–6. doi: 10.3109/13816810.2015.1005317 . 25675348

[8] Wang Y, Wei W, Zhang C, Zhang X, Liu M, Zhu X, et al. Association of Interleukin-1 Gene Single Nucleotide Polymorphisms with Keratoconus in Chinese Han Population. Curr Eye Res. 2016 May;41(5):630–5. doi: 10.3109/02713683.2015.1045083 . 26200829

[9] Wang Y, Jin T, Zhang X, Wei W, Cui Y, Geng T, et al. Common single nucleotide polymorphisms and keratoconus in the Han Chinese population. Ophthalmic Genet. 2013 Sep;34(3):160–6. doi: 10.3109/13816810.2012.743569 . 23289806

[10] Lechner J, Bae HA, Guduric-Fuchs J, Rice A, Govindarajan G, Siddiqui S, et al. Mutational analysis of MIR184 in sporadic keratoconus and myopia. Invest Ophthalmol Vis Sci. 2013 Aug 5;54(8):5266–72. doi: 10.1167/iovs.13-12035 . 23833072

[11] Guan T, Liu C, Ma Z, Ding S. The point mutation and polymorphism in keratoconus candidate gene TGFBI in Chinese population. Gene. 2012 Jul 15;503(1):137–9. doi: 10.1016/j.gene.2012.04.061 . 22575726

[12] Yildiz E, Bardak H, Gunay M, Bardak Y, Imamoglu S, Ozbas H, et al. Novel Zinc Finger Protein Gene 469 (ZNF469) Variants in Advanced Keratoconus. Curr Eye Res. 2017 Oct;42(10):1396–400. doi: 10.1080/02713683.2017.1325910 . 28622062

[13] Nemet AY, Vinker S, Bahar I, Kaiserman I. The association of keratoconus with immune disorders. Cornea. 2010 Nov;29(11):1261–4. doi: 10.1097/ICO.0b013e3181cb410b . 20802320

[14] Kim WJ, Rabinowitz YS, Meisler DM, Wilson SE. Keratocyte apoptosis associated with keratoconus. Exp Eye Res. 1999 Nov;69(5):475–81. doi: 10.1006/exer.1999.0719 . 10548467

[15] Fukuchi T, Yue BY, Sugar J, Lam S. Lysosomal enzyme activities in conjunctival tissues of patients with keratoconus. Arch Ophthalmol. 1994 Oct;112(10):1368–74. doi: 10.1001/archopht.1994.01090220118033 . 7945042

[16] Lema I, Duran JA. Inflammatory molecules in the tears of patients with keratoconus. Ophthalmology. 2005 Apr;112(4):654–9. doi: 10.1016/j.ophtha.2004.11.050 . 15808258

[17] Buddi R, Lin B, Atilano SR, Zorapapel NC, Kenney MC, Brown DJ. Evidence of oxidative stress in human corneal diseases. J Histochem Cytochem. 2002 Mar;50(3):341–51. doi: 10.1177/002215540205000306 . 11850437

[18] Shetty R, Sharma A, Pahuja N, Chevour P, Padmajan N, Dhamodaran K, et al. Oxidative stress induces dysregulated autophagy in corneal epithelium of keratoconus patients. PLoS One. 2017;12(9):e0184628. doi: 10.1371/journal.pone.0184628 . Pubmed Central PMCID: PMC5597215. 28902914 PMC5597215

[19] Bilgihan K, Hondur A, Sul S, Ozturk S. Pregnancy-induced progression of keratoconus. Cornea. 2011 Sep;30(9):991–4. doi: 10.1097/ICO.0b013e3182068adc . 21705880

[20] Galvis V, Tello A, Barrera R, Nino CA. Inflammation in Keratoconus. Cornea. 2015 Aug;34(8):e22–3. doi: 10.1097/ICO.0000000000000499 . 26075462

[21] Droitcourt C, Touboul D, Ged C, Ezzedine K, Cario-Andre M, de Verneuil H, et al. A prospective study of filaggrin null mutations in keratoconus patients with or without atopic disorders. Dermatology. 2011;222(4):336–41. doi: 10.1159/000328408 . 21701148

[22] Reinstein DZ, Archer TJ, Gobbe M. Corneal epithelial thickness profile in the diagnosis of keratoconus. Journal of refractive surgery. 2009 Jul;25(7):604–10. doi: 10.3928/1081597X-20090610-06 . 19662917

[23] Kanellopoulos AJ, Aslanides IM, Asimellis G. Correlation between epithelial thickness in normal corneas, untreated ectatic corneas, and ectatic corneas previously treated with CXL; is overall epithelial thickness a very early ectasia prognostic factor? Clin Ophthalmol. 2012;6:789–800. doi: 10.2147/OPTH.S31524 . Pubmed Central PMCID: PMC3373227. 22701079 PMC3373227

[24] Mace M, Galiacy SD, Erraud A, Mejia JE, Etchevers H, Allouche M, et al. Comparative transcriptome and network biology analyses demonstrate antiproliferative and hyperapoptotic phenotypes in human keratoconus corneas. Invest Ophthalmol Vis Sci. 2011 Aug 3;52(9):6181–91. doi: 10.1167/iovs.10-70981 . 21676910

[25] Henry J, Toulza E, Hsu CY, Pellerin L, Balica S, Mazereeuw-Hautier J, et al. Update on the epidermal differentiation complex. Front Biosci (Landmark Ed). 2012 Jan 1;17:1517–32. doi: 10.2741/4001 . 22201818

[26] Vandesompele J, De Preter K, Pattyn F, Poppe B, Van Roy N, De Paepe A, et al. Accurate normalization of real-time quantitative RT-PCR data by geometric averaging of multiple internal control genes. Genome Biol. 2002 Jun 18;3(7):RESEARCH0034. doi: 10.1186/gb-2002-3-7-research0034 . Pubmed Central PMCID: PMC126239. 12184808 PMC126239

[27] Guindolet D, Crouzet E, He Z, Herbepin P, Jumelle C, Perrache C, et al. Storage of Porcine Cornea in an Innovative Bioreactor. Invest Ophthalmol Vis Sci. 2017 Nov 1;58(13):5907–17. doi: 10.1167/iovs.17-22218 . 29164231

[28] Efron N, Hollingsworth JG. New perspectives on keratoconus as revealed by corneal confocal microscopy. Clin Exp Optom. 2008 Jan;91(1):34–55. doi: 10.1111/j.1444-0938.2007.00195.x . 18045250

[29] Tong L, Corrales RM, Chen Z, Villarreal AL, De Paiva CS, Beuerman R, et al. Expression and regulation of cornified envelope proteins in human corneal epithelium. Invest Ophthalmol Vis Sci. 2006 May;47(5):1938–46. doi: 10.1167/iovs.05-1129 . Pubmed Central PMCID: PMC2906387. 16639001 PMC2906387

[30] Simon M, Haftek M, Sebbag M, Montezin M, Girbal-Neuhauser E, Schmitt D, et al. Evidence that filaggrin is a component of cornified cell envelopes in human plantar epidermis. The Biochemical journal. 1996 Jul 1;317 (Pt 1):173–7. Pubmed Central PMCID: 1217460. doi: 10.1042/bj3170173 8694761 PMC1217460

[31] McGuire J, Osber M, Lightfoot L. Two keratins MW 50,000 and 56,000 are synthesized by psoriatic epidermis. Br J Dermatol. 1984 Jul;111 Suppl 27:27–37. doi: 10.1111/j.1365-2133.1984.tb15579.x . 6204677

[32] Schermer A, Jester JV, Hardy C, Milano D, Sun TT. Transient synthesis of K6 and K16 keratins in regenerating rabbit corneal epithelium: keratin markers for an alternative pathway of keratinocyte differentiation. Differentiation. 1989 Dec;42(2):103–10. doi: 10.1111/j.1432-0436.1989.tb00611.x . 2483836

[33] Chaerkady R, Shao H, Scott SG, Pandey A, Jun AS, Chakravarti S. The keratoconus corneal proteome: loss of epithelial integrity and stromal degeneration. J Proteomics. 2013 Jul 11;87:122–31. doi: 10.1016/j.jprot.2013.05.023 . Pubmed Central PMCID: PMC3721369. 23727491 PMC3721369

[34] Joseph R, Srivastava OP, Pfister RR. Differential epithelial and stromal protein profiles in keratoconus and normal human corneas. Exp Eye Res. 2011 Apr;92(4):282–98. doi: 10.1016/j.exer.2011.01.008 . 21281627

[35] Kalinin A, Marekov LN, Steinert PM. Assembly of the epidermal cornified cell envelope. J Cell Sci. 2001 Sep;114(Pt 17):3069–70. doi: 10.1242/jcs.114.17.3069 . 11590230

[36] Yoshida S, Yasuda M, Miyashita H, Ogawa Y, Yoshida T, Matsuzaki Y, et al. Generation of stratified squamous epithelial progenitor cells from mouse induced pluripotent stem cells. PLoS One. 2011;6(12):e28856. doi: 10.1371/journal.pone.0028856 . Pubmed Central PMCID: 3235161. 22174914 PMC3235161

[37] Schafer M, Farwanah H, Willrodt AH, Huebner AJ, Sandhoff K, Roop D, et al. Nrf2 links epidermal barrier function with antioxidant defense. EMBO Mol Med. 2012 May;4(5):364–79. doi: 10.1002/emmm.201200219 . Pubmed Central PMCID: PMC3403295. 22383093 PMC3403295

[38] Pradervand S, Yasukawa H, Muller OG, Kjekshus H, Nakamura T, St Amand TR, et al. Small proline-rich protein 1A is a gp130 pathway- and stress-inducible cardioprotective protein. EMBO J. 2004 Nov 10;23(22):4517–25. doi: 10.1038/sj.emboj.7600454 . Pubmed Central PMCID: PMC526469. 15510217 PMC526469

[39] Vermeij WP, Backendorf C. Skin cornification proteins provide global link between ROS detoxification and cell migration during wound healing. PLoS One. 2010 Aug 3;5(8):e11957. doi: 10.1371/journal.pone.0011957 . Pubmed Central PMCID: PMC2914756. 20689819 PMC2914756

[40] You J, Corley SM, Wen L, Hodge C, Hollhumer R, Madigan MC, et al. RNA-Seq analysis and comparison of corneal epithelium in keratoconus and myopia patients. Sci Rep. 2018 Jan 10;8(1):389. doi: 10.1038/s41598-017-18480-x . Pubmed Central PMCID: PMC5762683. 29321650 PMC5762683

[41] Loboda A, Damulewicz M, Pyza E, Jozkowicz A, Dulak J. Role of Nrf2/HO-1 system in development, oxidative stress response and diseases: an evolutionarily conserved mechanism. Cell Mol Life Sci. 2016 Sep;73(17):3221–47. doi: 10.1007/s00018-016-2223-0 . Pubmed Central PMCID: PMC4967105. 27100828 PMC4967105

[42] Poss KD, Tonegawa S. Reduced stress defense in heme oxygenase 1-deficient cells. Proc Natl Acad Sci U S A. 1997 Sep 30;94(20):10925–30. doi: 10.1073/pnas.94.20.10925 . Pubmed Central PMCID: PMC23533. 9380736 PMC23533

[43] Avetisov SE, Mamikonyan VR, Novikov IA, Pateyuk LS, Osipyan GA, Kiryushchenkova NP. [Abnormal distribution of trace elements in keratoconic corneas]. Vestn Oftalmol. 2015 Nov-Dec;131(6):34–42. doi: 10.17116/oftalma2015131634-42 . Pereraspredelenie mineral’nykh elementov v rogovitse pri keratokonuse. 26977725

[44] Shinde V, Hu N, Mahale A, Maiti G, Daoud Y, Eberhart CG, et al. RNA sequencing of corneas from two keratoconus patient groups identifies potential biomarkers and decreased NRF2-antioxidant responses. Sci Rep. 2020 Jun 18;10(1):9907. doi: 10.1038/s41598-020-66735-x . Pubmed Central PMCID: 7303170. 32555404 PMC7303170

[45] Stachon T, Latta L, Seitz B, Szentmary N. Hypoxic stress increases NF-kappaB and iNOS mRNA expression in normal, but not in keratoconus corneal fibroblasts. Graefe’s archive for clinical and experimental ophthalmology = Albrecht von Graefes Archiv fur klinische und experimentelle Ophthalmologie. 2021 Feb;259(2):449–58. doi: 10.1007/s00417-020-04900-8 . Pubmed Central PMCID: 7843574. 32886165 PMC7843574

[46] Lopez-Lopez M, Regueiro U, Bravo SB, Chantada-Vazquez MDP, Varela-Fernandez R, Avila-Gomez P, et al. Tear Proteomics in Keratoconus: A Quantitative SWATH-MS Analysis. Invest Ophthalmol Vis Sci. 2021 Aug 2;62(10):30. doi: 10.1167/iovs.62.10.30 . 34431975 PMC8399462

[47] Kerns M, DePianto D, Yamamoto M, Coulombe PA. Differential modulation of keratin expression by sulforaphane occurs via Nrf2-dependent and -independent pathways in skin epithelia. Molecular biology of the cell. 2010 Dec;21(23):4068–75. doi: 10.1091/mbc.E10-02-0153 . Pubmed Central PMCID: 2993737. 20926689 PMC2993737

[48] Monteiro de Barros MR, Chakravarti S. Pathogenesis of keratoconus: NRF2-antioxidant, extracellular matrix and cellular dysfunctions. Exp Eye Res. 2022 Apr 3;219:109062. doi: 10.1016/j.exer.2022.109062 . 35385756 PMC12056795

