# Supplementary figures and images for: Corneal epithelium in keratoconus underexpresses active NRF2 and a subset of oxidative stress-related genes
Source: PLoS One. 2022 Oct 14;17(10):e0273807. doi: 10.1371/journal.pone.0273807 (PMC9565379; doi:10.1371/journal.pone.0273807)

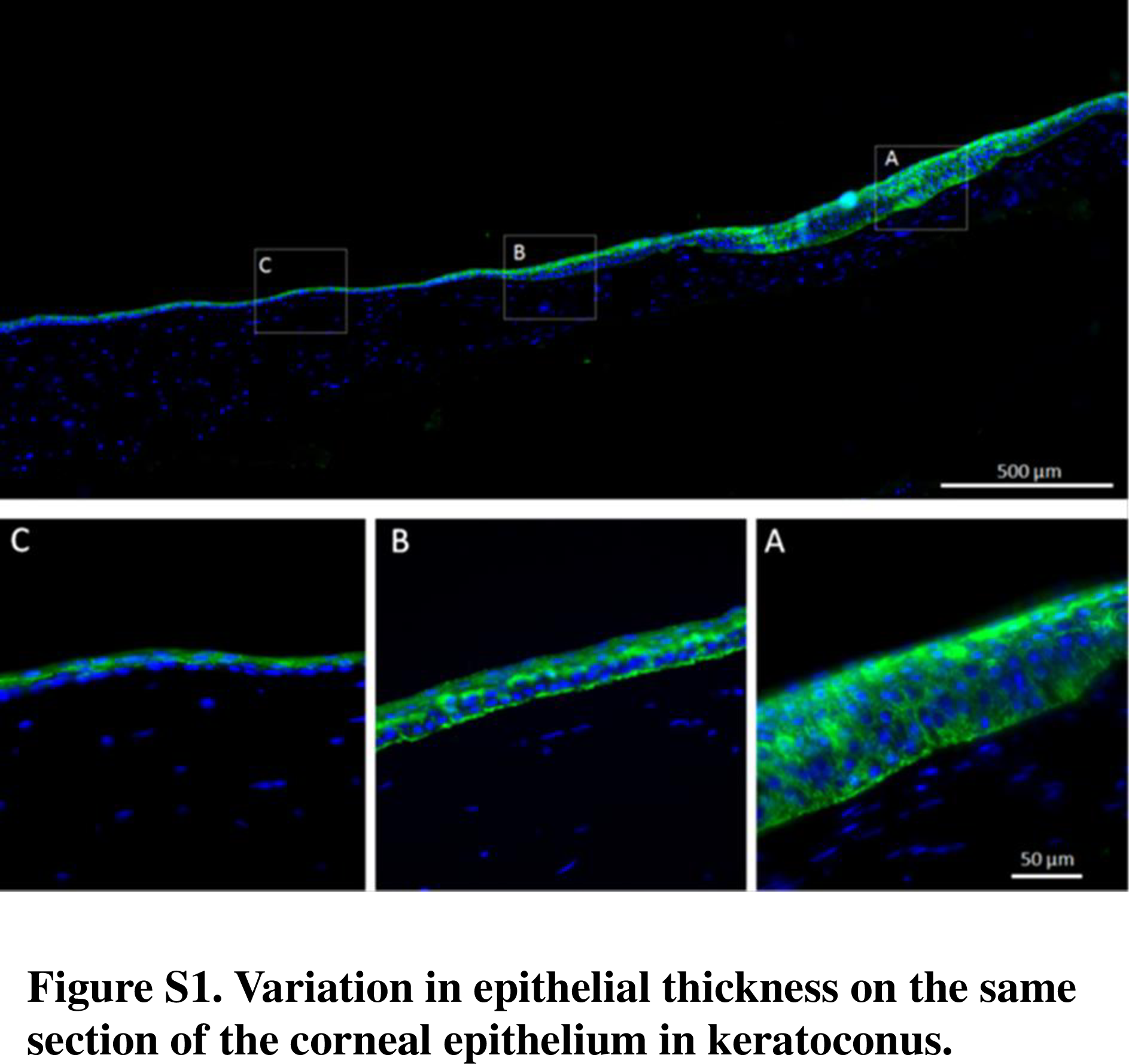

Supplement: S1 Fig — Representative immunodetection of KRT3 (green) on frozen sections of human corneal epithelium of keratoconus patients (n = 3). Nuclei were stained with TO-PRO Iodide (blue). A-C, Enlargements of the indicated areas. A: peripheral hyperplasic zone, B: intermediate zone, normal thickness, C: corneal apex hypoplasic zone. (TIF) [file pone.0273807.s001.tif]

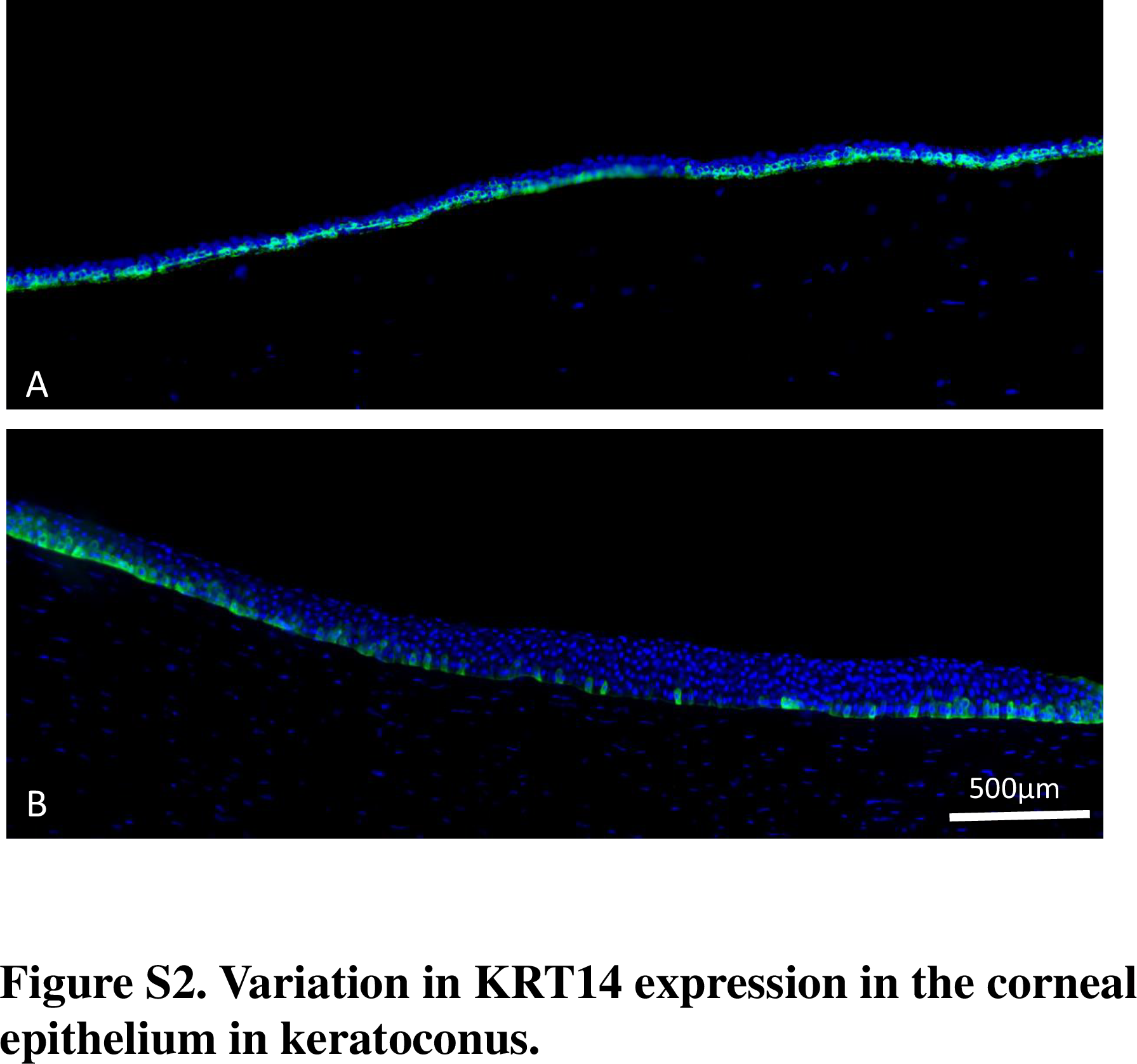

Supplement: S2 Fig — Representative immunodetection of KRT14 (green) on frozen sections of human corneal epithelium of keratoconus versus control patients. Nuclei were stained with DAPI (blue). A: control patient; B: keratoconus patient. (TIF) [file pone.0273807.s002.tif]

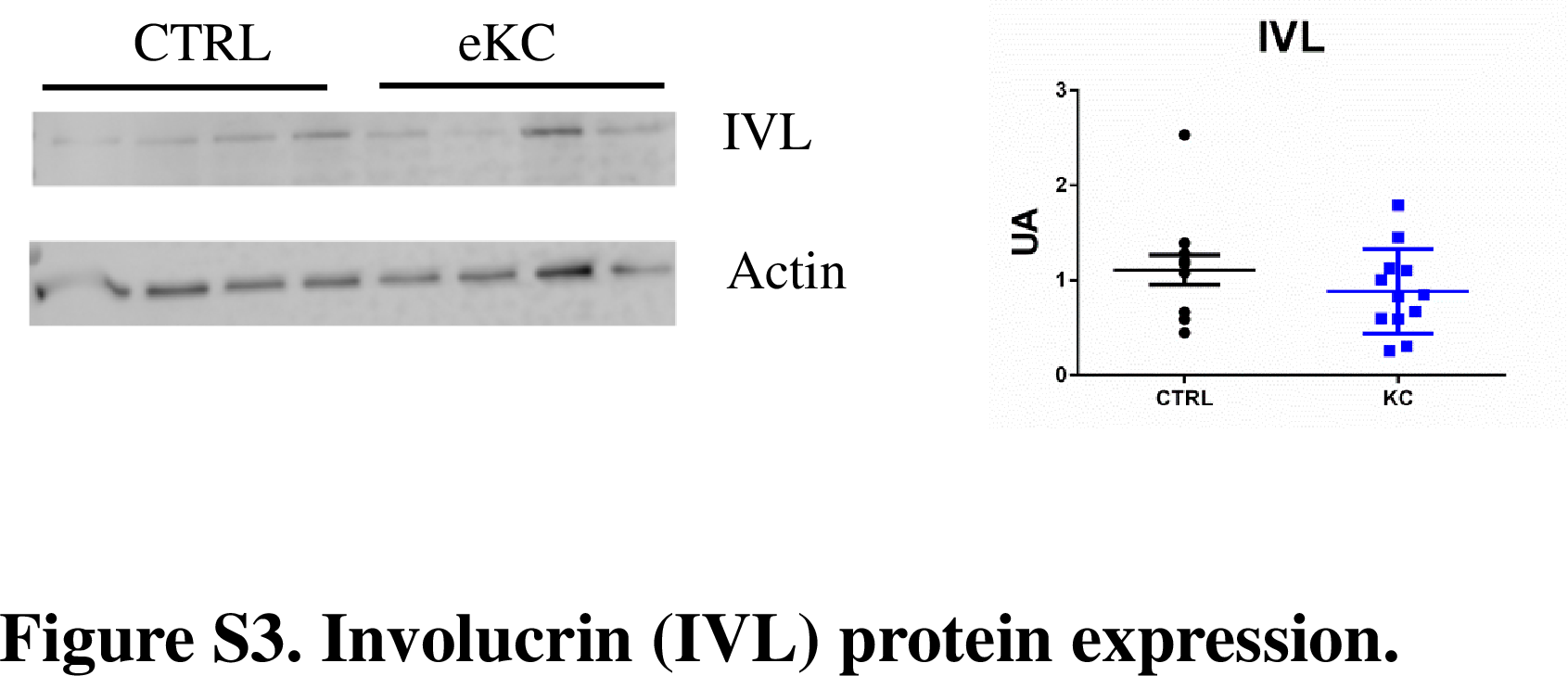

Supplement: S3 Fig — Proteins of control (CTRL) and early keratoconus (eKC) samples were immunoblotted with antibodies specific for IVL and actin, respectively (one representative blot with 4 samples of each condition). Densitometry quantification of western-blots using Image J; n = 12, P = 0.27. (TIF) [file pone.0273807.s003.tif]

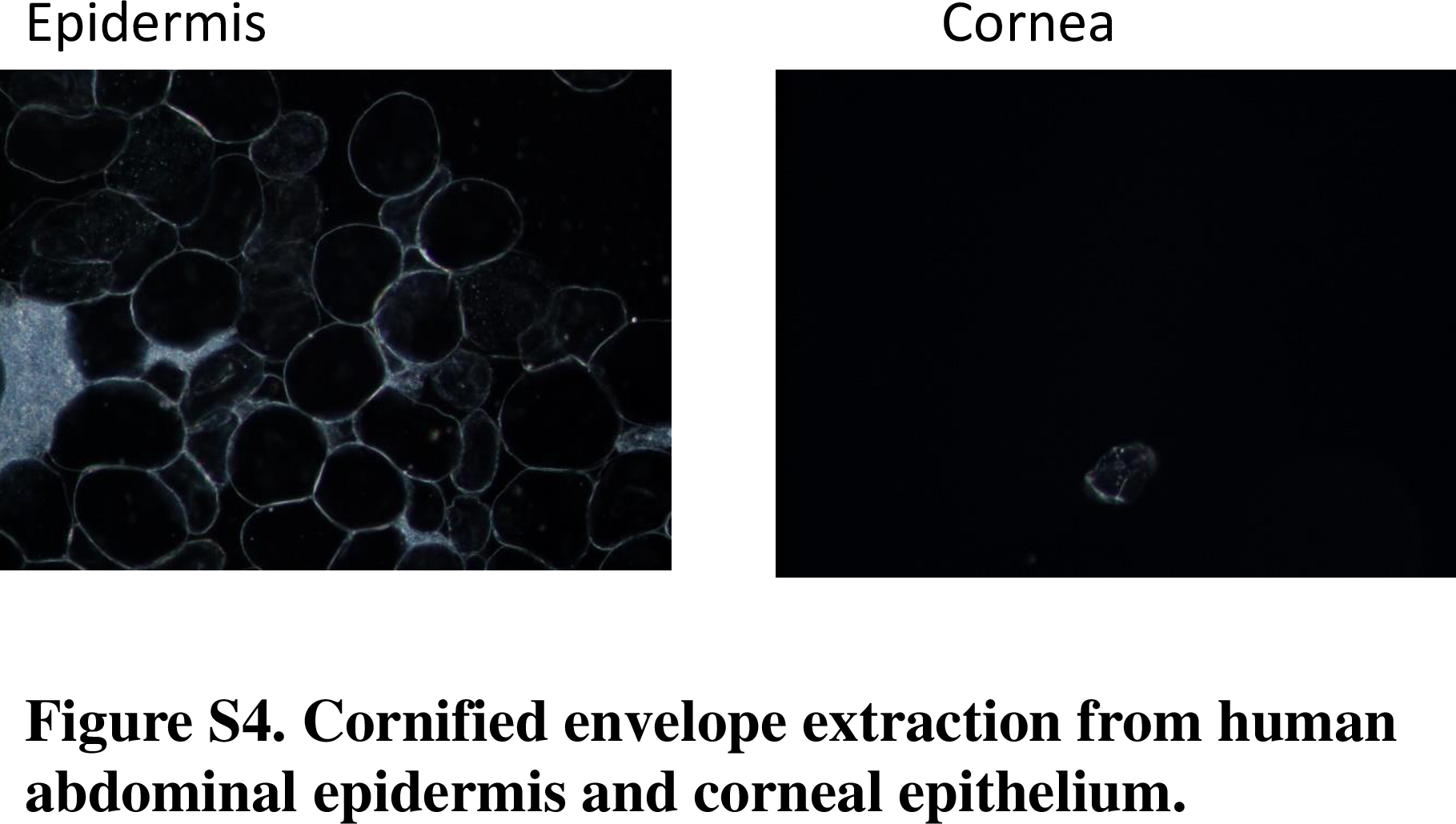

Supplement: S4 Fig — Optical microscope observation. X20 magnification. (TIF) [file pone.0273807.s004.tif]

IVL

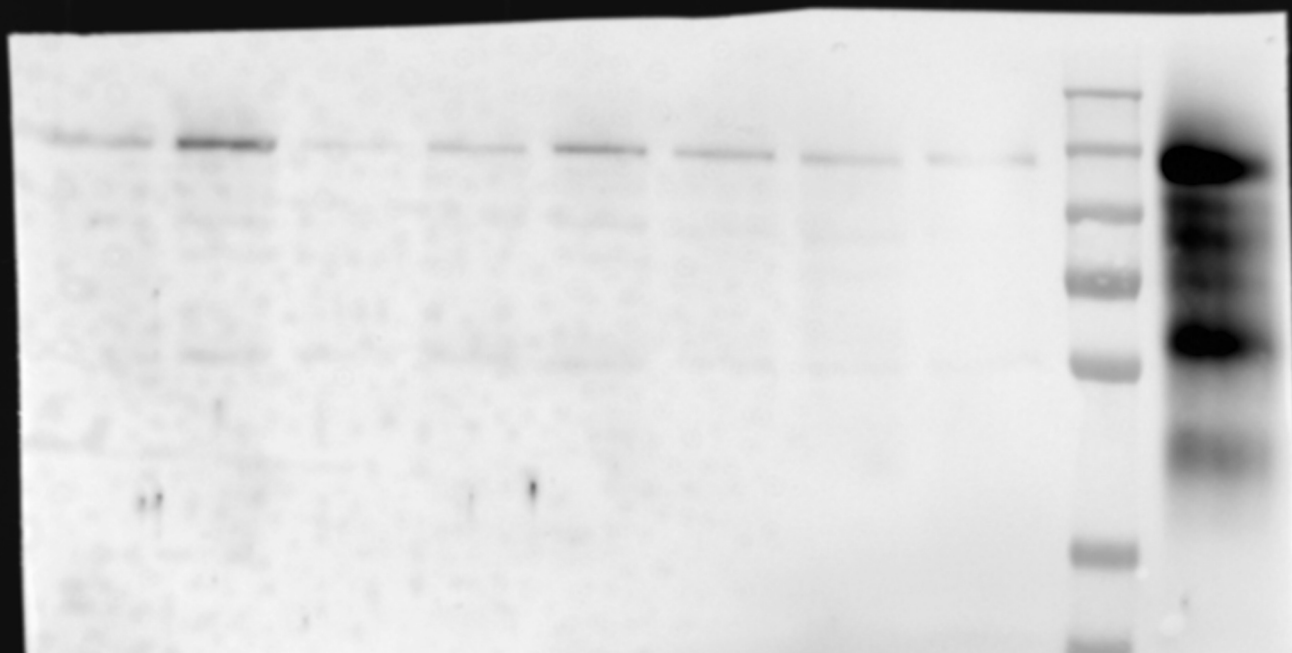

KC samples lane 1-4

Control samples lane 5-8

MW

X

positive  
epiderm  
control

actin

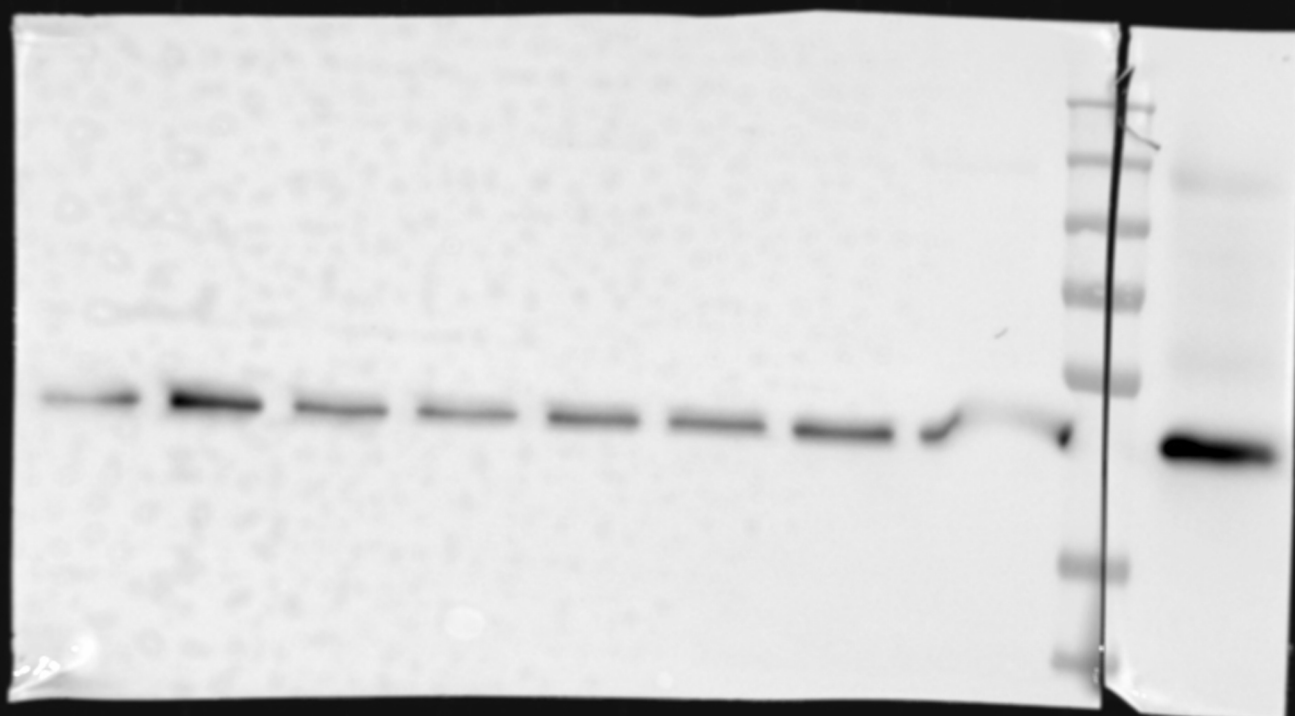

MW

X

Supplement: S1 Raw images — (PDF) [file pone.0273807.s008.pdf]
