# Supplementary material for: Corneal epithelium in keratoconus underexpresses active NRF2 and a subset of oxidative stress-related genes
Source: PLoS One. 2022 Oct 14;17(10):e0273807. doi: 10.1371/journal.pone.0273807 (PMC9565379; doi:10.1371/journal.pone.0273807)
Supplement: S1 Methods — (DOCX) [file pone.0273807.s005.docx]

**Supplementary Methods 1**

**Immunoblotting analysis**

Corneal epithelial tissues from control and early KC group (n= 12 for each group) were collected as described in previous section and stored in liquid nitrogen.

Soluble proteins were extracted in TE-NP40 buffer (40 mM Tris-HCl pH 7.5, 0.5% Nonidet-P40, 10 mM EDTA, and 0.25 mM PMSF) for 15min using a homogenizer, followed by centrifugation at 12,000 × g for 15 min at 4 °C. The supernatant was removed, aliquoted and stored at −80 °C until use.

Equal amounts of protein (according to Ponceau staining) were resolved by SDS-10% polyacrylamide gel electrophoresis and electrotransfered onto nitrocellulose membranes (GE Healthcare, Danderyd, Sweden). Membranes were blocked in PBS 1X-Tween 0.2% containing 5% skimmed milk, then incubated overnight at 4°C with anti-IVL (clone SY5, Sigma-Aldrich) diluted at 1:10000 or anti-beta actin at 1:10000 (A3854, Sigma-Aldrich) and finely incubated for 2h at room temperature with secondary horseradish peroxidase-conjugated antibodies diluted to 2:10000 (Goat anti-Mouse IgG-HRP; Bethyl Laboratories, Montgomery, TX; Goat Anti-rabbit IgG-HRP; Southern Biotech, Birmingham, AL). The detection was realized with ECL Prime system (GE Healthcare) and images acquired with a G:BOX Chemi XT4CCD camera (Syngene, Cambridge, United Kingdom) and GeneSys software (Genesys, Daly City, CA). ImageJ software was used to quantify immunoreactive bands. Signals were normalized to actin immunodetection.
