# Supplementary material for: Corneal epithelium in keratoconus underexpresses active NRF2 and a subset of oxidative stress-related genes
Source: PLoS One. 2022 Oct 14;17(10):e0273807. doi: 10.1371/journal.pone.0273807 (PMC9565379; doi:10.1371/journal.pone.0273807)
Supplement: S1 Table — (DOCX) [file pone.0273807.s006.docx]

**Supplementary Table S1. Sequences of primers used for qPCR.**

| Gene name | Forward (5’-3’) | Reverse (5’-3’) |
| --- | --- | --- |
| *B2M* | GAGGCTATCCAGCGTACTCCA | CGGCAGGCATACTCATCTTTT |
| *CDSN* | ACTGCTGCTGGCTGGTCT | AGAGCTTCTGGCACTGGAAA |
| *CLDN 1* | TGGCATGAAGTGTATGAAGTGCTT | AGAGCTTCTGGCACTGGAAA |
| *CUL3* | TGTGGAGAACGTCTACAATTTGG | GCGCCTCTGTCTACGACTT |
| *DSC1* | CATGGGTGGTCAGCCTTTCGGT | TCCTGATCCTGTACCTTCATTGGCA |
| *DSG1* | GAAGGCAGAAACGTGAATGGA | TTTTGGCGATTGGGTTCCT |
| *DSG2* | CTAACAGGTTACGCTTTGGATGC | GTGAACACTGGTTCGTTGTCAT |
| *DSG3* | GCAAAAACGTGAATGGGTGAAA | TCCAGAGATTCGGTAGGTGATT |
| *DSP* | GCA-GGA-TGT-ACT-ATT-CTC-GGC | CCT-GGA-TGG-TGT-TCT-GGT-TCT |
| *EVPL* | GGCGAAGCTCAACTCCAACTT | CAGCCGTTTTTCCTCTGCC |
| *FLG* | GCAAGGTCAAGTCCAGGAGAA | CCCTCGGTTTCCACTGTCTC |
| *FLG2* | TCTGAAGAACCCAGATGATCCA | CATCAAAAGAAACTCAGTAAAGTCCAA |
| *HMOX1* | AAGACTGCGTTCCTGCTCAAC | AAAGCCCTACAGCAACTGTCG |
| *HMOX2* | TCAGCGGAAGTGGAAACCTC | AGAAGTCCTTGACAAACTGGGT |
| *HRNR* | AGGACAGGGCTATAGTCAGCA | CCGAAGCGTGATGGGAGG |
| *IVL* | GGGTGGTTATTTATGTTTGGGTGG | GCCAGGTCCAAGACATTCAAC |
| *KEAP1* | CTGGAGGATCATACCAAGCAGG | GGATACCCTCAATGGACACCAC |
| *KRT3* | GCAGGGCACAAGTTCCATCT | TCTCTCCCCGAGGATGTTGTC |
| *KRT10* | TGATGTGAATGTGGAAATGAATGC | GTAGTCAGTTCCTTGCTCTTTTCA |
| *KRT16* | GACCGGCGGAGATGTGAAC | CTGCTCGTACTGGTCACGC |
| *LOR* | CGAAGGAGTTGGAGGTGTTT | ACTGGGGTTGGGAGGTAGTT |
| *LOX* | CGGCGGAGGAAAACTGTCT | TCGGCTGGGTAAGAAATCTGA |
| *NQO1* | GAAGAGCACTGATCGTACTGGC | GGATACTGAAAGTTCGCAGGG |
| *NRF2* | TCAGCGACGGAAAGAGTATGA | CCACTGGTTTCTGACTGGATGT |
| *PPL* | GCACCAATGAGCTGTACTGG | GCTGGGGTAGTCGAGGTTG |
| *SPRR1A* | TGGCCACTGGATACTGAACA | CCCAAATCCATCCTCAAATG |
| *SPRR2A* | TATTTGGCTCACCTCGTTCC | CCAGGACTTCCTTTGCTCAG |
| *SPRR3* | TTCCACAACCTGGAAACACA | TTCAGGGACCTTGGTGTAGC |
| *TBP* | CCACTCACAGACTCTCACAAC | CTGCGGTACAATCCCAGAACT |
| *TGM1* | CCCCCGCAATGAGATCTACA | ATCCTCATGGTCCACGTACACA |
| *TGM3* | GGAAGGACTCTGCCACAATGTC | TGTCTGATTCAGGTACTTCTCATACTG |
| *TGM5* | CGGAGCAGGTTGAGGACTGT | GAGGACTCCAAGGAAGACTTTCTG |
| *UBB* | GGTCCTGCGTCTGAGAGGT | GGCCTTCACATTTTCGATGGT |
