# Supplementary material for: Corneal epithelium in keratoconus underexpresses active NRF2 and a subset of oxidative stress-related genes
Source: PLoS One. 2022 Oct 14;17(10):e0273807. doi: 10.1371/journal.pone.0273807 (PMC9565379; doi:10.1371/journal.pone.0273807)
Supplement: S2 Table — (DOCX) [file pone.0273807.s007.docx]

**Supplementary Table S2.** Differential expression analysis of different subgroups of patient.

| Gene name | Global ddCT | Atopic ddCT | Non atopic ddCT |
| --- | --- | --- | --- |
| *KRT16* | 3,49 | 3,38 | 3,58 |
| *KRT14* | 1,67 | 1,93 | 1,57 |
| *SPRR1A* | 2,28 | 2,05 | 2,02 |
| *SPRR2A* | 3,19 | 4,03 | 2,31 |
| *TGM1* | 1,62 | 1,23 | 0,91 |
| *NRF2* | 1,13 | 0,3 | 1,51 |
| *HMOX1* | 2,84 | 3,09 | 2,64 |
| *HMOX2* | 1,27 | 0,3 | 1,94 |

ddCT: delta delta Cycle threshold normalized to that of geometric mean of housekeeping genes.
